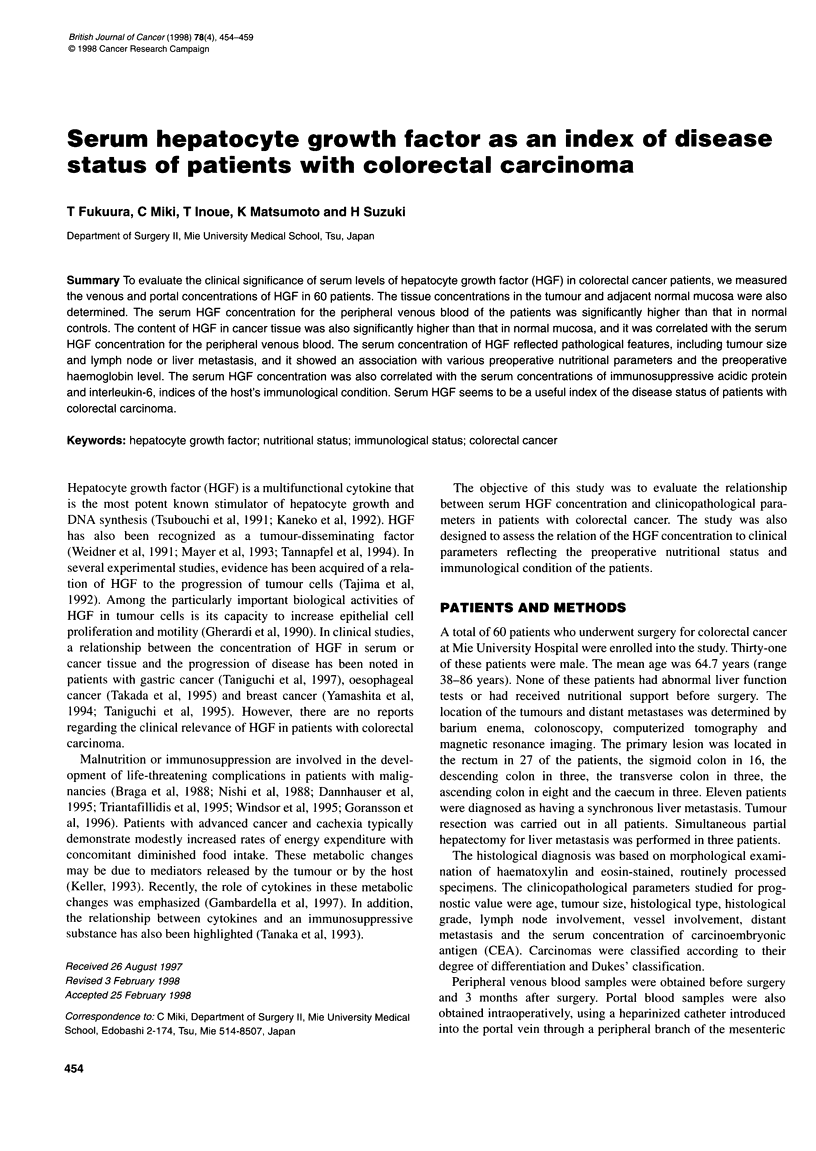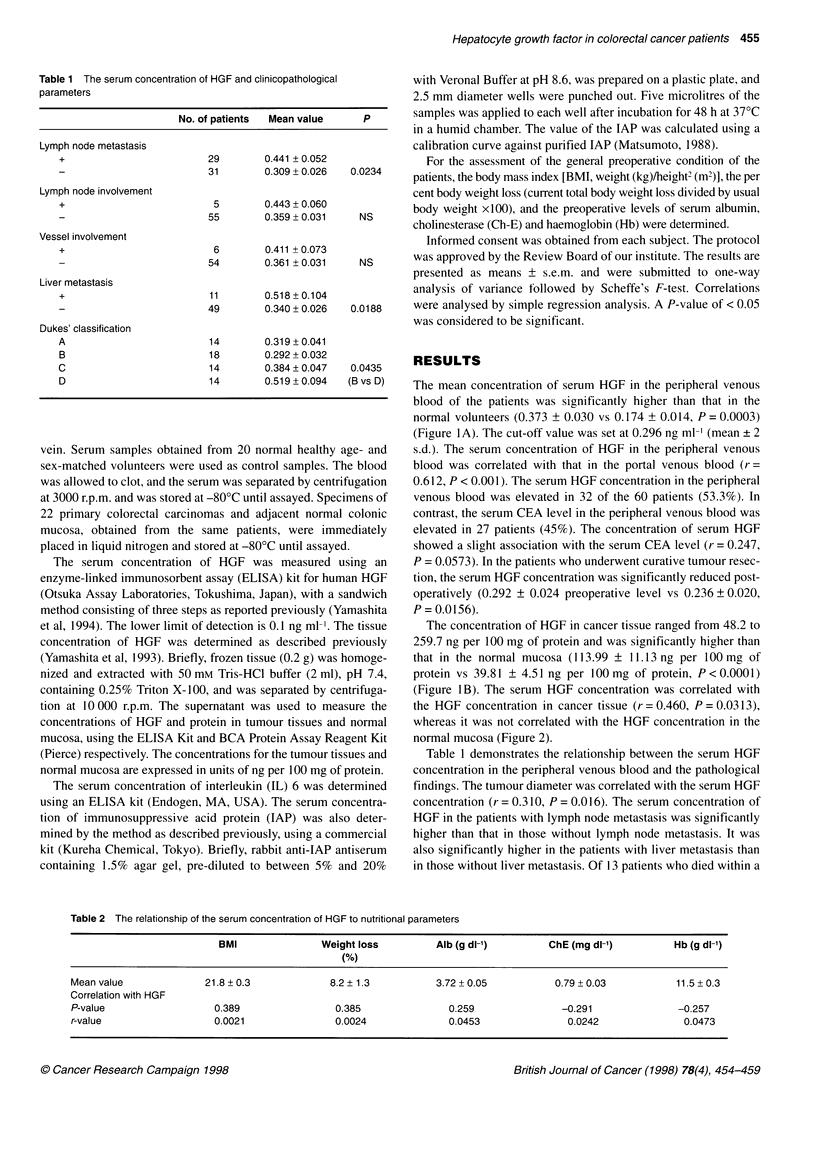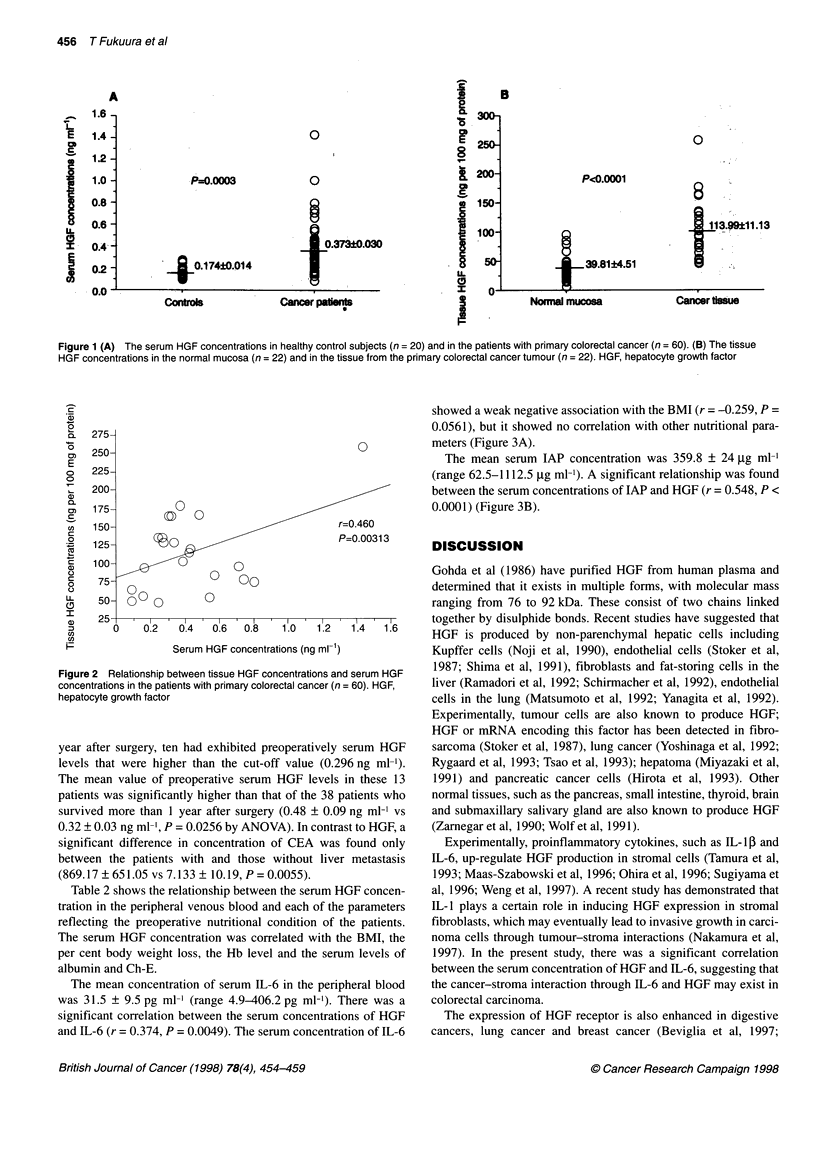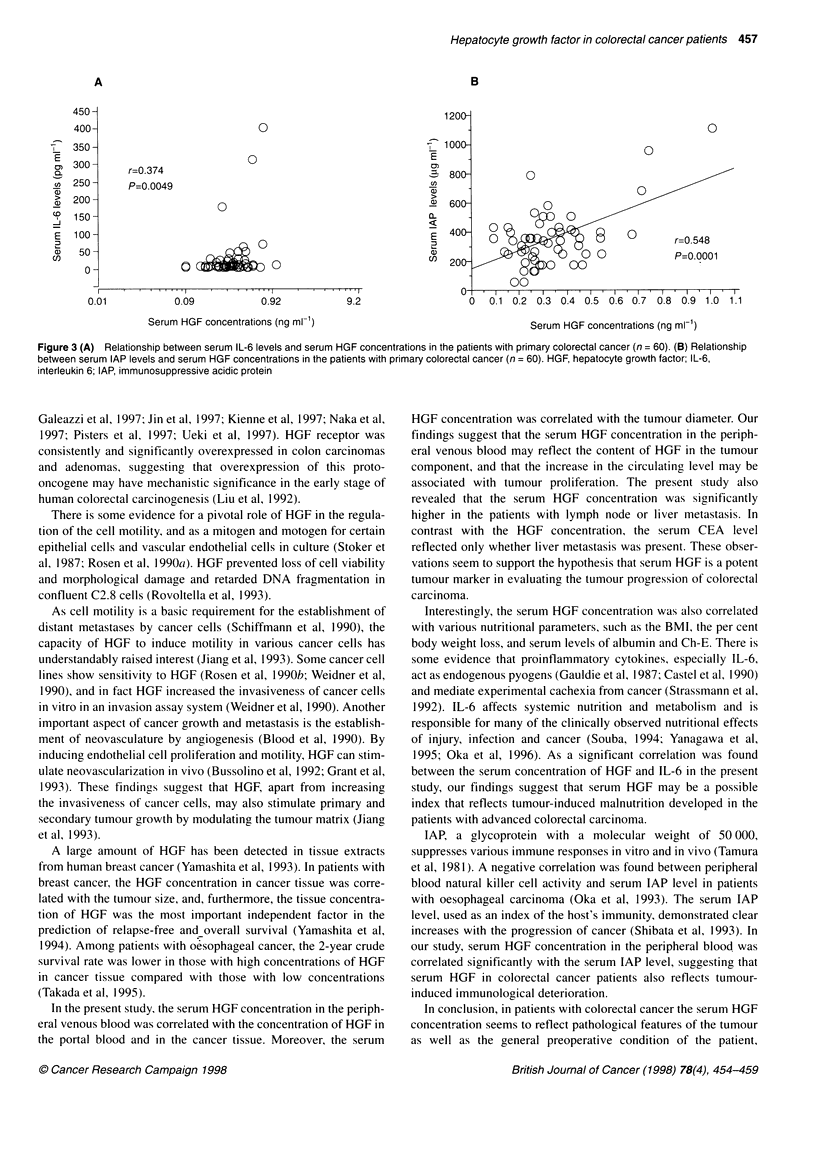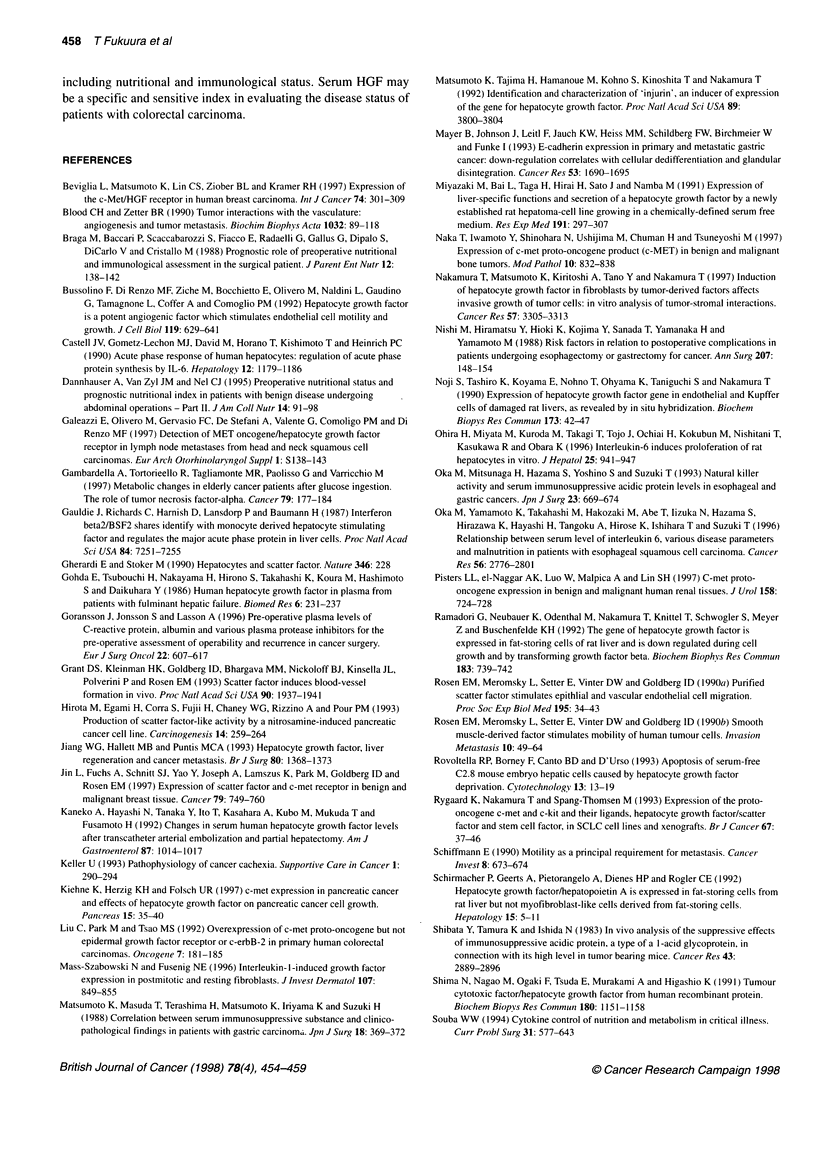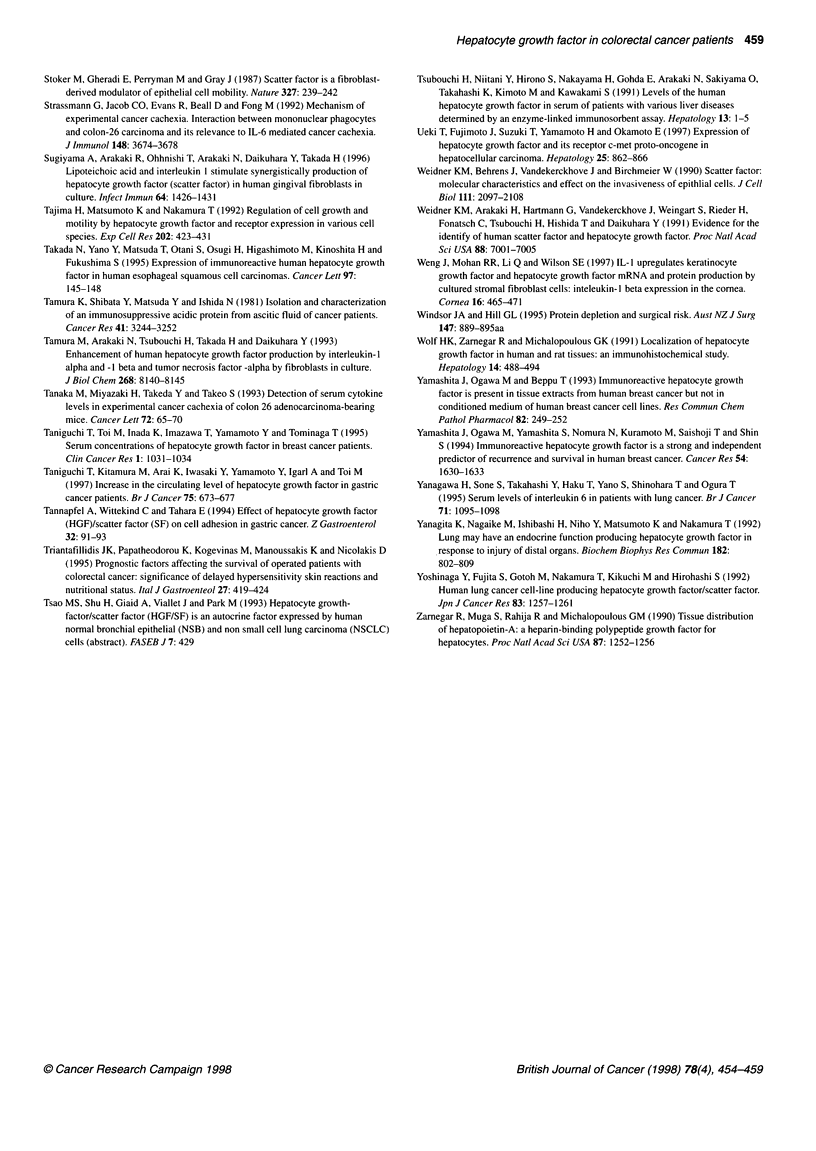# Serum hepatocyte growth factor as an index of disease status of patients with colorectal carcinoma.

**DOI:** 10.1038/bjc.1998.514

**Published:** 1998-08

**Authors:** T. Fukuura, C. Miki, T. Inoue, K. Matsumoto, H. Suzuki

**Affiliations:** Department of Surgery II, Mie University Medical School, Tsu, Japan.

## Abstract

To evaluate the clinical significance of serum levels of hepatocyte growth factor (HGF) in colorectal cancer patients, we measured the venous and portal concentrations of HGF in 60 patients. The tissue concentrations in the tumour and adjacent normal mucosa were also determined. The serum HGF concentration for the peripheral venous blood of the patients was significantly higher than that in normal controls. The content of HGF in cancer tissue was also significantly higher than that in normal mucosa, and it was correlated with the serum HGF concentration for the peripheral venous blood. The serum concentration of HGF reflected pathological features, including tumour size and lymph node or liver metastasis, and it showed an association with various preoperative nutritional parameters and the preoperative haemoglobin level. The serum HGF concentration was also correlated with the serum concentrations of immunosuppressive acidic protein and interleukin-6, indices of the host's immunological condition. Serum HGF seems to be a useful index of the disease status of patients with colorectal carcinoma.


British Journal of Cancer (1998) 78(4), 454–459
© 1998 Cancer Research Campaign

### Serum hepatocyte growth factor as an index of disease status of patients with colorectal carcinoma

**T Fukuura, C Miki, T Inoue, K Matsumoto and H Suzuki**

Department of Surgery II, Mie University Medical School, Tsu, Japan

**Summary** To evaluate the clinical significance of serum levels of hepatocyte growth factor (HGF) in colorectal cancer patients, we measured the venous and portal concentrations of HGF in 60 patients. The tissue concentrations in the tumour and adjacent normal mucosa were also determined. The serum HGF concentration for the peripheral venous blood of the patients was significantly higher than that in normal controls. The content of HGF in cancer tissue was also significantly higher than that in normal mucosa, and it was correlated with the serum HGF concentration for the peripheral venous blood. The serum concentration of HGF reflected pathological features, including tumour size and lymph node or liver metastasis, and it showed an association with various preoperative nutritional parameters and the preoperative haemoglobin level. The serum HGF concentration was also correlated with the serum concentrations of immunosuppressive acidic protein and interleukin-6, indices of the host's immunological condition. Serum HGF seems to be a useful index of the disease status of patients with colorectal carcinoma.

**Keywords:** hepatocyte growth factor; nutritional status; immunological status; colorectal cancer

Hepatocyte growth factor (HGF) is a multifunctional cytokine that is the most potent known stimulator of hepatocyte growth and DNA synthesis (Tsubouchi et al, 1991; Kaneko et al, 1992). HGF has also been recognized as a tumour-disseminating factor (Weidner et al, 1991; Mayer et al, 1993; Tannapfel et al, 1994). In several experimental studies, evidence has been acquired of a relation of HGF to the progression of tumour cells (Tajima et al, 1992). Among the particularly important biological activities of HGF in tumour cells is its capacity to increase epithelial cell proliferation and motility (Gherardi et al, 1990). In clinical studies, a relationship between the concentration of HGF in serum or cancer tissue and the progression of disease has been noted in patients with gastric cancer (Taniguchi et al, 1997), oesophageal cancer (Takada et al, 1995) and breast cancer (Yamashita et al, 1994; Taniguchi et al, 1995). However, there are no reports regarding the clinical relevance of HGF in patients with colorectal carcinoma.

Malnutrition or immunosuppression are involved in the development of life-threatening complications in patients with malignancies (Braga et al, 1988; Nishi et al, 1988; Dannhauser et al, 1995; Triantafillidis et al, 1995; Windsor et al, 1995; Goransson et al, 1996). Patients with advanced cancer and cachexia typically demonstrate modestly increased rates of energy expenditure with concomitant diminished food intake. These metabolic changes may be due to mediators released by the tumour or by the host (Keller, 1993). Recently, the role of cytokines in these metabolic changes was emphasized (Gambardella et al, 1997). In addition, the relationship between cytokines and an immunosuppressive substance has also been highlighted (Tanaka et al, 1993).

*Received 26 August 1997*
*Revised 3 February 1998*
*Accepted 25 February 1998*

*Correspondence to:* C Miki, Department of Surgery II, Mie University Medical School, Edobashi 2-174, Tsu, Mie 514-8507, Japan

The objective of this study was to evaluate the relationship between serum HGF concentration and clinicopathological parameters in patients with colorectal cancer. The study was also designed to assess the relation of the HGF concentration to clinical parameters reflecting the preoperative nutritional status and immunological condition of the patients.

### PATIENTS AND METHODS

A total of 60 patients who underwent surgery for colorectal cancer at Mie University Hospital were enrolled into the study. Thirty-one of these patients were male. The mean age was 64.7 years (range 38–86 years). None of these patients had abnormal liver function tests or had received nutritional support before surgery. The location of the tumours and distant metastases was determined by barium enema, colonoscopy, computerized tomography and magnetic resonance imaging. The primary lesion was located in the rectum in 27 of the patients, the sigmoid colon in 16, the descending colon in three, the transverse colon in three, the ascending colon in eight and the caecum in three. Eleven patients were diagnosed as having a synchronous liver metastasis. Tumour resection was carried out in all patients. Simultaneous partial hepatectomy for liver metastasis was performed in three patients.

The histological diagnosis was based on morphological examination of haematoxylin and eosin-stained, routinely processed specimens. The clinicopathological parameters studied for prognostic value were age, tumour size, histological type, histological grade, lymph node involvement, vessel involvement, distant metastasis and the serum concentration of carcinoembryonic antigen (CEA). Carcinomas were classified according to their degree of differentiation and Dukes' classification.

Peripheral venous blood samples were obtained before surgery and 3 months after surgery. Portal blood samples were also obtained intraoperatively, using a heparinized catheter introduced into the portal vein through a peripheral branch of the mesenteric

**Table 1** The serum concentration of HGF and clinicopathological parameters

| | No. of patients | Mean value | *P* |
|---|---|---|---|
| Lymph node metastasis | | | |
| + | 29 | 0.441 ± 0.052 | |
| – | 31 | 0.309 ± 0.026 | 0.0234 |
| Lymph node involvement | | | |
| + | 5 | 0.443 ± 0.060 | |
| – | 55 | 0.359 ± 0.031 | NS |
| Vessel involvement | | | |
| + | 6 | 0.411 ± 0.073 | |
| – | 54 | 0.361 ± 0.031 | NS |
| Liver metastasis | | | |
| + | 11 | 0.518 ± 0.104 | |
| – | 49 | 0.340 ± 0.026 | 0.0188 |
| Dukes' classification | | | |
| A | 14 | 0.319 ± 0.041 | |
| B | 18 | 0.292 ± 0.032 | |
| C | 14 | 0.384 ± 0.047 | 0.0435 |
| D | 14 | 0.519 ± 0.094 | (B vs D) |

vein. Serum samples obtained from 20 normal healthy age- and sex-matched volunteers were used as control samples. The blood was allowed to clot, and the serum was separated by centrifugation at 3000 r.p.m. and was stored at –80°C until assayed. Specimens of 22 primary colorectal carcinomas and adjacent normal colonic mucosa, obtained from the same patients, were immediately placed in liquid nitrogen and stored at –80°C until assayed.

The serum concentration of HGF was measured using an enzyme-linked immunosorbent assay (ELISA) kit for human HGF (Otsuka Assay Laboratories, Tokushima, Japan), with a sandwich method consisting of three steps as reported previously (Yamashita et al, 1994). The lower limit of detection is 0.1 ng $ml^{-1}$. The tissue concentration of HGF was determined as described previously (Yamashita et al, 1993). Briefly, frozen tissue (0.2 g) was homogenized and extracted with 50 mM Tris-HCl buffer (2 ml), pH 7.4, containing 0.25% Triton X-100, and was separated by centrifugation at 10 000 r.p.m. The supernatant was used to measure the concentrations of HGF and protein in tumour tissues and normal mucosa, using the ELISA Kit and BCA Protein Assay Reagent Kit (Pierce) respectively. The concentrations for the tumour tissues and normal mucosa are expressed in units of ng per 100 mg of protein.

The serum concentration of interleukin (IL) 6 was determined using an ELISA kit (Endogen, MA, USA). The serum concentration of immunosuppressive acid protein (IAP) was also determined by the method as described previously, using a commercial kit (Kureha Chemical, Tokyo). Briefly, rabbit anti-IAP antiserum containing 1.5% agar gel, pre-diluted to between 5% and 20% with Veronal Buffer at pH 8.6, was prepared on a plastic plate, and 2.5 mm diameter wells were punched out. Five microlitres of the samples was applied to each well after incubation for 48 h at 37°C in a humid chamber. The value of the IAP was calculated using a calibration curve against purified IAP (Matsumoto, 1988).

For the assessment of the general preoperative condition of the patients, the body mass index [BMI, weight (kg)/height$^2$ (m$^2$)], the per cent body weight loss (current total body weight loss divided by usual body weight ×100), and the preoperative levels of serum albumin, cholinesterase (Ch-E) and haemoglobin (Hb) were determined.

Informed consent was obtained from each subject. The protocol was approved by the Review Board of our institute. The results are presented as means ± s.e.m. and were submitted to one-way analysis of variance followed by Scheffe's *F*-test. Correlations were analysed by simple regression analysis. A *P*-value of < 0.05 was considered to be significant.

### RESULTS

The mean concentration of serum HGF in the peripheral venous blood of the patients was significantly higher than that in the normal volunteers (0.373 ± 0.030 vs 0.174 ± 0.014, $P = 0.0003$) (Figure 1A). The cut-off value was set at 0.296 ng $ml^{-1}$ (mean ± 2 s.d.). The serum concentration of HGF in the peripheral venous blood was correlated with that in the portal venous blood ($r = 0.612$, $P < 0.001$). The serum HGF concentration in the peripheral venous blood was elevated in 32 of the 60 patients (53.3%). In contrast, the serum CEA level in the peripheral venous blood was elevated in 27 patients (45%). The concentration of serum HGF showed a slight association with the serum CEA level ($r = 0.247$, $P = 0.0573$). In the patients who underwent curative tumour resection, the serum HGF concentration was significantly reduced postoperatively (0.292 ± 0.024 preoperative level vs 0.236 ± 0.020, $P = 0.0156$).

The concentration of HGF in cancer tissue ranged from 48.2 to 259.7 ng per 100 mg of protein and was significantly higher than that in the normal mucosa (113.99 ± 11.13 ng per 100 mg of protein vs 39.81 ± 4.51 ng per 100 mg of protein, $P < 0.0001$) (Figure 1B). The serum HGF concentration was correlated with the HGF concentration in cancer tissue ($r = 0.460$, $P = 0.0313$), whereas it was not correlated with the HGF concentration in the normal mucosa (Figure 2).

Table 1 demonstrates the relationship between the serum HGF concentration in the peripheral venous blood and the pathological findings. The tumour diameter was correlated with the serum HGF concentration ($r = 0.310$, $P = 0.016$). The serum concentration of HGF in the patients with lymph node metastasis was significantly higher than that in those without lymph node metastasis. It was also significantly higher in the patients with liver metastasis than in those without liver metastasis. Of 13 patients who died within a

**Table 2** The relationship of the serum concentration of HGF to nutritional parameters

| | BMI | Weight loss (%) | Alb (g $dl^{-1}$) | ChE (mg $dl^{-1}$) | Hb (g $dl^{-1}$) |
|---|---|---|---|---|---|
| Mean value | 21.8 ± 0.3 | 8.2 ± 1.3 | 3.72 ± 0.05 | 0.79 ± 0.03 | 11.5 ± 0.3 |
| Correlation with HGF | | | | | |
| *P*-value | 0.389 | 0.385 | 0.259 | –0.291 | –0.257 |
| *r*-value | 0.0021 | 0.0024 | 0.0453 | 0.0242 | 0.0473 |

**Figure 1 (A)** The serum HGF concentrations in healthy control subjects ($n$ = 20) and in the patients with primary colorectal cancer ($n$ = 60). **(B)** The tissue HGF concentrations in the normal mucosa ($n$ = 22) and in the tissue from the primary colorectal cancer tumour ($n$ = 22). HGF, hepatocyte growth factor

**Figure 2** Relationship between tissue HGF concentrations and serum HGF concentrations in the patients with primary colorectal cancer ($n$ = 60). HGF, hepatocyte growth factor year after surgery, ten had exhibited preoperatively serum HGF levels that were higher than the cut-off value (0.296 ng ml$^{-1}$). The mean value of preoperative serum HGF levels in these 13 patients was significantly higher than that of the 38 patients who survived more than 1 year after surgery (0.48 ± 0.09 ng ml$^{-1}$ vs 0.32 ± 0.03 ng ml$^{-1}$, $P$ = 0.0256 by ANOVA). In contrast to HGF, a significant difference in concentration of CEA was found only between the patients with and those without liver metastasis (869.17 ± 651.05 vs 7.133 ± 10.19, $P$ = 0.0055).

Table 2 shows the relationship between the serum HGF concentration in the peripheral venous blood and each of the parameters reflecting the preoperative nutritional condition of the patients. The serum HGF concentration was correlated with the BMI, the per cent body weight loss, the Hb level and the serum levels of albumin and Ch-E.

The mean concentration of serum IL-6 in the peripheral blood was 31.5 ± 9.5 pg ml$^{-1}$ (range 4.9–406.2 pg ml$^{-1}$). There was a significant correlation between the serum concentrations of HGF and IL-6 ($r$ = 0.374, $P$ = 0.0049). The serum concentration of IL-6 showed a weak negative association with the BMI ($r$ = –0.259, $P$ = 0.0561), but it showed no correlation with other nutritional parameters (Figure 3A).

The mean serum IAP concentration was 359.8 ± 24 μg ml$^{-1}$ (range 62.5–1112.5 μg ml$^{-1}$). A significant relationship was found between the serum concentrations of IAP and HGF ($r$ = 0.548, $P$ < 0.0001) (Figure 3B).

### DISCUSSION

Gohda et al (1986) have purified HGF from human plasma and determined that it exists in multiple forms, with molecular mass ranging from 76 to 92 kDa. These consist of two chains linked together by disulphide bonds. Recent studies have suggested that HGF is produced by non-parenchymal hepatic cells including Kupffer cells (Noji et al, 1990), endothelial cells (Stoker et al, 1987; Shima et al, 1991), fibroblasts and fat-storing cells in the liver (Ramadori et al, 1992; Schirmacher et al, 1992), endothelial cells in the lung (Matsumoto et al, 1992; Yanagita et al, 1992). Experimentally, tumour cells are also known to produce HGF; HGF or mRNA encoding this factor has been detected in fibrosarcoma (Stoker et al, 1987), lung cancer (Yoshinaga et al, 1992; Rygaard et al, 1993; Tsao et al, 1993); hepatoma (Miyazaki et al, 1991) and pancreatic cancer cells (Hirota et al, 1993). Other normal tissues, such as the pancreas, small intestine, thyroid, brain and submaxillary salivary gland are also known to produce HGF (Zarnegar et al, 1990; Wolf et al, 1991).

Experimentally, proinflammatory cytokines, such as IL-1β and IL-6, up-regulate HGF production in stromal cells (Tamura et al, 1993; Maas-Szabowski et al, 1996; Ohira et al, 1996; Sugiyama et al, 1996; Weng et al, 1997). A recent study has demonstrated that IL-1 plays a certain role in inducing HGF expression in stromal fibroblasts, which may eventually lead to invasive growth in carcinoma cells through tumour–stroma interactions (Nakamura et al, 1997). In the present study, there was a significant correlation between the serum concentration of HGF and IL-6, suggesting that the cancer–stroma interaction through IL-6 and HGF may exist in colorectal carcinoma.

The expression of HGF receptor is also enhanced in digestive cancers, lung cancer and breast cancer (Beviglia et al, 1997;

© Cancer Research Campaign 1998

**Figure 3 (A)** Relationship between serum IL-6 levels and serum HGF concentrations in the patients with primary colorectal cancer (*n* = 60). **(B)** Relationship between serum IAP levels and serum HGF concentrations in the patients with primary colorectal cancer (*n* = 60). HGF, hepatocyte growth factor; IL-6, interleukin 6; IAP, immunosuppressive acidic protein

Galeazzi et al, 1997; Jin et al, 1997; Kienne et al, 1997; Naka et al, 1997; Pisters et al, 1997; Ueki et al, 1997). HGF receptor was consistently and significantly overexpressed in colon carcinomas and adenomas, suggesting that overexpression of this proto-oncogene may have mechanistic significance in the early stage of human colorectal carcinogenesis (Liu et al, 1992).

There is some evidence for a pivotal role of HGF in the regulation of the cell motility, and as a mitogen and motogen for certain epithelial cells and vascular endothelial cells in culture (Stoker et al, 1987; Rosen et al, 1990*a*). HGF prevented loss of cell viability and morphological damage and retarded DNA fragmentation in confluent C2.8 cells (Rovoltella et al, 1993).

As cell motility is a basic requirement for the establishment of distant metastases by cancer cells (Schiffmann et al, 1990), the capacity of HGF to induce motility in various cancer cells has understandably raised interest (Jiang et al, 1993). Some cancer cell lines show sensitivity to HGF (Rosen et al, 1990*b*; Weidner et al, 1990), and in fact HGF increased the invasiveness of cancer cells in vitro in an invasion assay system (Weidner et al, 1990). Another important aspect of cancer growth and metastasis is the establishment of neovasculature by angiogenesis (Blood et al, 1990). By inducing endothelial cell proliferation and motility, HGF can stimulate neovascularization in vivo (Bussolino et al, 1992; Grant et al, 1993). These findings suggest that HGF, apart from increasing the invasiveness of cancer cells, may also stimulate primary and secondary tumour growth by modulating the tumour matrix (Jiang et al, 1993).

A large amount of HGF has been detected in tissue extracts from human breast cancer (Yamashita et al, 1993). In patients with breast cancer, the HGF concentration in cancer tissue was correlated with the tumour size, and, furthermore, the tissue concentration of HGF was the most important independent factor in the prediction of relapse-free and overall survival (Yamashita et al, 1994). Among patients with oesophageal cancer, the 2-year crude survival rate was lower in those with high concentrations of HGF in cancer tissue compared with those with low concentrations (Takada et al, 1995).

In the present study, the serum HGF concentration in the peripheral venous blood was correlated with the concentration of HGF in the portal blood and in the cancer tissue. Moreover, the serum HGF concentration was correlated with the tumour diameter. Our findings suggest that the serum HGF concentration in the peripheral venous blood may reflect the content of HGF in the tumour component, and that the increase in the circulating level may be associated with tumour proliferation. The present study also revealed that the serum HGF concentration was significantly higher in the patients with lymph node or liver metastasis. In contrast with the HGF concentration, the serum CEA level reflected only whether liver metastasis was present. These observations seem to support the hypothesis that serum HGF is a potent tumour marker in evaluating the tumour progression of colorectal carcinoma.

Interestingly, the serum HGF concentration was also correlated with various nutritional parameters, such as the BMI, the per cent body weight loss, and serum levels of albumin and Ch-E. There is some evidence that proinflammatory cytokines, especially IL-6, act as endogenous pyogens (Gauldie et al, 1987; Castel et al, 1990) and mediate experimental cachexia from cancer (Strassmann et al, 1992). IL-6 affects systemic nutrition and metabolism and is responsible for many of the clinically observed nutritional effects of injury, infection and cancer (Souba, 1994; Yanagawa et al, 1995; Oka et al, 1996). As a significant correlation was found between the serum concentration of HGF and IL-6 in the present study, our findings suggest that serum HGF may be a possible index that reflects tumour-induced malnutrition developed in the patients with advanced colorectal carcinoma.

IAP, a glycoprotein with a molecular weight of 50 000, suppresses various immune responses in vitro and in vivo (Tamura et al, 1981). A negative correlation was found between peripheral blood natural killer cell activity and serum IAP level in patients with oesophageal carcinoma (Oka et al, 1993). The serum IAP level, used as an index of the host's immunity, demonstrated clear increases with the progression of cancer (Shibata et al, 1993). In our study, serum HGF concentration in the peripheral blood was correlated significantly with the serum IAP level, suggesting that serum HGF in colorectal cancer patients also reflects tumour-induced immunological deterioration.

In conclusion, in patients with colorectal cancer the serum HGF concentration seems to reflect pathological features of the tumour as well as the general preoperative condition of the patient,

© Cancer Research Campaign 1998

including nutritional and immunological status. Serum HGF may be a specific and sensitive index in evaluating the disease status of patients with colorectal carcinoma.

### REFERENCES

Beviglia L, Matsumoto K, Lin CS, Ziober BL and Kramer RH (1997) Expression of the c-Met/HGF receptor in human breast carcinoma. *Int J Cancer* **74**: 301–309

Blood CH and Zetter BR (1990) Tumor interactions with the vasculature: angiogenesis and tumor metastasis. *Biochim Biophys Acta* **1032**: 89–118

Braga M, Baccari P, Scaccabarozzi S, Fiacco E, Radaelli G, Gallus G, Dipalo S, DiCarlo V and Cristallo M (1988) Prognostic role of preoperative nutritional and immunological assessment in the surgical patient. *J Parent Ent Nutr* **12**: 138–142

Bussolino F, Di Renzo MF, Ziche M, Bocchietto E, Olivero M, Naldini L, Gaudino G, Tamagnone L, Coffer A and Comoglio PM (1992) Hepatocyte growth factor is a potent angiogenic factor which stimulates endothelial cell motility and growth. *J Cell Biol* **119**: 629–641

Castell JV, Gometz-Lechon MJ, David M, Horano T, Kishimoto T and Heinrich PC (1990) Acute phase response of human hepatocytes: regulation of acute phase protein synthesis by IL-6. *Hepatology* **12**: 1179–1186

Dannhauser A, Van Zyl JM and Nel CJ (1995) Preoperative nutritional status and prognostic nutritional index in patients with benign disease undergoing abdominal operations – Part II. *J Am Coll Nutr* **14**: 91–98

Galeazzi E, Olivero M, Gervasio FC, De Stefani A, Valente G, Comoligo PM and Di Renzo MF (1997) Detection of MET oncogene/hepatocyte growth factor receptor in lymph node metastases from head and neck squamous cell carcinomas. *Eur Arch Otorhinolaryngol Suppl* **1**: S138–143

Gambardella A, Tortorieello R, Tagliamonte MR, Paolisso G and Varricchio M (1997) Metabolic changes in elderly cancer patients after glucose ingestion. The role of tumor necrosis factor-alpha. *Cancer* **79**: 177–184

Gauldie J, Richards C, Harnish D, Lansdorp P and Baumann H (1987) Interferon beta2/BSF2 shares identify with monocyte derived hepatocyte stimulating factor and regulates the major acute phase protein in liver cells. *Proc Natl Acad Sci USA* **84**: 7251–7255

Gherardi E and Stoker M (1990) Hepatocytes and scatter factor. *Nature* **346**: 228

Gohda E, Tsubouchi H, Nakayama H, Hirono S, Takahashi K, Koura M, Hashimoto S and Daikuhara Y (1986) Human hepatocyte growth factor in plasma from patients with fulminant hepatic failure. *Biomed Res* **6**: 231–237

Goransson J, Jonsson S and Lasson A (1996) Pre-operative plasma levels of C-reactive protein, albumin and various plasma protease inhibitors for the pre-operative assessment of operability and recurrence in cancer surgery. *Eur J Surg Oncol* **22**: 607–617

Grant DS, Kleinman HK, Goldberg ID, Bhargava MM, Nickoloff BJ, Kinsella JL, Polverini P and Rosen EM (1993) Scatter factor induces blood-vessel formation in vivo. *Proc Natl Acad Sci USA* **90**: 1937–1941

Hirota M, Egami H, Corra S, Fujii H, Chaney WG, Rizzino A and Pour PM (1993) Production of scatter factor-like activity by a nitrosamine-induced pancreatic cancer cell line. *Carcinogenesis* **14**: 259–264

Jiang WG, Hallett MB and Puntis MCA (1993) Hepatocyte growth factor, liver regeneration and cancer metastasis. *Br J Surg* **80**: 1368–1373

Jin L, Fuchs A, Schnitt SJ, Yao Y, Joseph A, Lamszus K, Park M, Goldberg ID and Rosen EM (1997) Expression of scatter factor and c-met receptor in benign and malignant breast tissue. *Cancer* **79**: 749–760

Kaneko A, Hayashi N, Tanaka Y, Ito T, Kasahara A, Kubo M, Mukuda T and Fusamoto H (1992) Changes in serum human hepatocyte growth factor levels after transcatheter arterial embolization and partial hepatectomy. *Am J Gastroenterol* **87**: 1014–1017

Keller U (1993) Pathophysiology of cancer cachexia. *Supportive Care in Cancer* **1**: 290–294

Kiehne K, Herzig KH and Folsch UR (1997) c-met expression in pancreatic cancer and effects of hepatocyte growth factor on pancreatic cancer cell growth. *Pancreas* **15**: 35–40

Liu C, Park M and Tsao MS (1992) Overexpression of c-met proto-oncogene but not epidermal growth factor receptor or c-erbB-2 in primary human colorectal carcinomas. *Oncogene* **7**: 181–185

Mass-Szabowski N and Fusenig NE (1996) Interleukin-1-induced growth factor expression in postmitotic and resting fibroblasts. *J Invest Dermatol* **107**: 849–855

Matsumoto K, Masuda T, Terashima H, Matsumoto K, Iriyama K and Suzuki H (1988) Correlation between serum immunosuppressive substance and clinicopathological findings in patients with gastric carcinoma. *Jpn J Surg* **18**: 369–372

Matsumoto K, Tajima H, Hamanoue M, Kohno S, Kinoshita T and Nakamura T (1992) Identification and characterization of 'injurin', an inducer of expression of the gene for hepatocyte growth factor. *Proc Natl Acad Sci USA* **89**: 3800–3804

Mayer B, Johnson J, Leitl F, Jauch KW, Heiss MM, Schildberg FW, Birchmeier W and Funke I (1993) E-cadherin expression in primary and metastatic gastric cancer: down-regulation correlates with cellular dedifferentiation and glandular disintegration. *Cancer Res* **53**: 1690–1695

Miyazaki M, Bai L, Taga H, Hirai H, Sato J and Namba M (1991) Expression of liver-specific functions and secretion of a hepatocyte growth factor by a newly established rat hepatoma-cell line growing in a chemically-defined serum free medium. *Res Exp Med* **191**: 297–307

Naka T, Iwamoto Y, Shinohara N, Ushijima M, Chuman H and Tsuneyoshi M (1997) Expression of c-met proto-oncogene product (c-MET) in benign and malignant bone tumors. *Mod Pathol* **10**: 832–838

Nakamura T, Matsumoto K, Kiritoshi A, Tano Y and Nakamura T (1997) Induction of hepatocyte growth factor in fibroblasts by tumor-derived factors affects invasive growth of tumor cells: in vitro analysis of tumor-stromal interactions. *Cancer Res* **57**: 3305–3313

Nishi M, Hiramatsu Y, Hioki K, Kojima Y, Sanada T, Yamanaka H and Yamamoto M (1988) Risk factors in relation to postoperative complications in patients undergoing esophagectomy or gastrectomy for cancer. *Ann Surg* **207**: 148–154

Noji S, Tashiro K, Koyama E, Nohno T, Ohyama K, Taniguchi S and Nakamura T (1990) Expression of hepatocyte growth factor gene in endothelial and Kupffer cells of damaged rat livers, as revealed by in situ hybridization. *Biochem Biopys Res Commun* **173**: 42–47

Ohira H, Miyata M, Kuroda M, Takagi T, Tojo J, Ochiai H, Kokubun M, Nishitani T, Kasukawa R and Obara K (1996) Interleukin-6 induces proloferation of rat hepatocytes in vitro. *J Hepatol* **25**: 941–947

Oka M, Mitsunaga H, Hazama S, Yoshino S and Suzuki T (1993) Natural killer activity and serum immunosuppressive acidic protein levels in esophageal and gastric cancers. *Jpn J Surg* **23**: 669–674

Oka M, Yamamoto K, Takahashi M, Hakozaki M, Abe T, Iizuka N, Hazama S, Hirazawa K, Hayashi H, Tangoku A, Hirose K, Ishihara T and Suzuki T (1996) Relationship between serum level of interleukin 6, various disease parameters and malnutrition in patients with esophageal squamous cell carcinoma. *Cancer Res* **56**: 2776–2801

Pisters LL, el-Naggar AK, Luo W, Malpica A and Lin SH (1997) C-met proto-oncogene expression in benign and malignant human renal tissues. *J Urol* **158**: 724–728

Ramadori G, Neubauer K, Odenthal M, Nakamura T, Knittel T, Schwogler S, Meyer Z and Buschenfelde KH (1992) The gene of hepatocyte growth factor is expressed in fat-storing cells of rat liver and is down regulated during cell growth and by transforming growth factor beta. *Biochem Biophys Res Commun* **183**: 739–742

Rosen EM, Meromsky L, Setter E, Vinter DW and Goldberg ID (1990*a*) Purified scatter factor stimulates epithlial and vascular endothelial cell migration. *Proc Soc Exp Biol Med* **195**: 34–43

Rosen EM, Meromsky L, Setter E, Vinter DW and Goldberg ID (1990*b*) Smooth muscle-derived factor stimulates mobility of human tumour cells. *Invasion Metastasis* **10**: 49–64

Rovoltella RP, Borney F, Canto BD and D'Urso (1993) Apoptosis of serum-free C2.8 mouse embryo hepatic cells caused by hepatocyte growth factor deprivation. *Cytotechnology* **13**: 13–19

Rygaard K, Nakamura T and Spang-Thomsen M (1993) Expression of the proto-oncogene c-met and c-kit and their ligands, hepatocyte growth factor/scatter factor and stem cell factor, in SCLC cell lines and xenografts. *Br J Cancer* **67**: 37–46

Schiffmann E (1990) Motility as a principal requirement for metastasis. *Cancer Invest* **8**: 673–674

Schirmacher P, Geerts A, Pietorangelo A, Dienes HP and Rogler CE (1992) Hepatocyte growth factor/hepatopoietin A is expressed in fat-storing cells from rat liver but not myofibroblast-like cells derived from fat-storing cells. *Hepatology* **15**: 5–11

Shibata Y, Tamura K and Ishida N (1983) In vivo analysis of the suppressive effects of immunosuppressive acidic protein, a type of a 1-acid glycoprotein, in connection with its high level in tumor bearing mice. *Cancer Res* **43**: 2889–2896

Shima N, Nagao M, Ogaki F, Tsuda E, Murakami A and Higashio K (1991) Tumour cytotoxic factor/hepatocyte growth factor from human recombinant protein. *Biochem Biopys Res Commun* **180**: 1151–1158

Souba WW (1994) Cytokine control of nutrition and metabolism in critical illness. *Curr Probl Surg* **31**: 577–643

© Cancer Research Campaign 1998

Stoker M, Gheradi E, Perryman M and Gray J (1987) Scatter factor is a fibroblast-derived modulator of epithelial cell mobility. *Nature* **327**: 239–242

Strassmann G, Jacob CO, Evans R, Beall D and Fong M (1992) Mechanism of experimental cancer cachexia. Interaction between mononuclear phagocytes and colon-26 carcinoma and its relevance to IL-6 mediated cancer cachexia. *J Immunol* **148**: 3674–3678

Sugiyama A, Arakaki R, Ohhnishi T, Arakaki N, Daikuhara Y, Takada H (1996) Lipoteichoic acid and interleukin 1 stimulate synergistically production of hepatocyte growth factor (scatter factor) in human gingival fibroblasts in culture. *Infect Immun* **64**: 1426–1431

Tajima H, Matsumoto K and Nakamura T (1992) Regulation of cell growth and motility by hepatocyte growth factor and receptor expression in various cell species. *Exp Cell Res* **202**: 423–431

Takada N, Yano Y, Matsuda T, Otani S, Osugi H, Higashimoto M, Kinoshita H and Fukushima S (1995) Expression of immunoreactive human hepatocyte growth factor in human esophageal squamous cell carcinomas. *Cancer Lett* **97**: 145–148

Tamura K, Shibata Y, Matsuda Y and Ishida N (1981) Isolation and characterization of an immunosuppressive acidic protein from ascitic fluid of cancer patients. *Cancer Res* **41**: 3244–3252

Tamura M, Arakaki N, Tsubouchi H, Takada H and Daikuhara Y (1993) Enhancement of human hepatocyte growth factor production by interleukin-1 alpha and -1 beta and tumor necrosis factor -alpha by fibroblasts in culture. *J Biol Chem* **268**: 8140–8145

Tanaka M, Miyazaki H, Takeda Y and Takeo S (1993) Detection of serum cytokine levels in experimental cancer cachexia of colon 26 adenocarcinoma-bearing mice. *Cancer Lett* **72**: 65–70

Taniguchi T, Toi M, Inada K, Imazawa T, Yamamoto Y and Tominaga T (1995) Serum concentrations of hepatocyte growth factor in breast cancer patients. *Clin Cancer Res* **1**: 1031–1034

Taniguchi T, Kitamura M, Arai K, Iwasaki Y, Yamamoto Y, Igarl A and Toi M (1997) Increase in the circulating level of hepatocyte growth factor in gastric cancer patients. *Br J Cancer* **75**: 673–677

Tannapfel A, Wittekind C and Tahara E (1994) Effect of hepatocyte growth factor (HGF)/scatter factor (SF) on cell adhesion in gastric cancer. *Z Gastroenterol* **32**: 91–93

Triantafillidis JK, Papatheodorou K, Kogevinas M, Manoussakis K and Nicolakis D (1995) Prognostic factors affecting the survival of operated patients with colorectal cancer: significance of delayed hypersensitivity skin reactions and nutritional status. *Ital J Gastroenteol* **27**: 419–424

Tsao MS, Shu H, Giaid A, Viallet J and Park M (1993) Hepatocyte growth-factor/scatter factor (HGF/SF) is an autocrine factor expressed by human normal bronchial epithelial (NSB) and non small cell lung carcinoma (NSCLC) cells (abstract). *FASEB J* **7**: 429

Tsubouchi H, Niitani Y, Hirono S, Nakayama H, Gohda E, Arakaki N, Sakiyama O, Takahashi K, Kimoto M and Kawakami S (1991) Levels of the human hepatocyte growth factor in serum of patients with various liver diseases determined by an enzyme-linked immunosorbent assay. *Hepatology* **13**: 1–5

Ueki T, Fujimoto J, Suzuki T, Yamamoto H and Okamoto E (1997) Expression of hepatocyte growth factor and its receptor c-met proto-oncogene in hepatocellular carcinoma. *Hepatology* **25**: 862–866

Weidner KM, Behrens J, Vandekerckhove J and Birchmeier W (1990) Scatter factor: molecular characteristics and effect on the invasiveness of epithlial cells. *J Cell Biol* **111**: 2097–2108

Weidner KM, Arakaki H, Hartmann G, Vandekerckhove J, Weingart S, Rieder H, Fonatsch C, Tsubouchi H, Hishida T and Daikuhara Y (1991) Evidence for the identify of human scatter factor and hepatocyte growth factor. *Proc Natl Acad Sci USA* **88**: 7001–7005

Weng J, Mohan RR, Li Q and Wilson SE (1997) IL-1 upregulates keratinocyte growth factor and hepatocyte growth factor mRNA and protein production by cultured stromal fibroblast cells: inteleukin-1 beta expression in the cornea. *Cornea* **16**: 465–471

Windsor JA and Hill GL (1995) Protein depletion and surgical risk. *Aust NZ J Surg* **147**: 889–895aa

Wolf HK, Zarnegar R and Michalopoulous GK (1991) Localization of hepatocyte growth factor in human and rat tissues: an immunohistochemical study. *Hepatology* **14**: 488–494

Yamashita J, Ogawa M and Beppu T (1993) Immunoreactive hepatocyte growth factor is present in tissue extracts from human breast cancer but not in conditioned medium of human breast cancer cell lines. *Res Commun Chem Pathol Pharmacol* **82**: 249–252

Yamashita J, Ogawa M, Yamashita S, Nomura N, Kuramoto M, Saishoji T and Shin S (1994) Immunoreactive hepatocyte growth factor is a strong and independent predictor of recurrence and survival in human breast cancer. *Cancer Res* **54**: 1630–1633

Yanagawa H, Sone S, Takahashi Y, Haku T, Yano S, Shinohara T and Ogura T (1995) Serum levels of interleukin 6 in patients with lung cancer. *Br J Cancer* **71**: 1095–1098

Yanagita K, Nagaike M, Ishibashi H, Niho Y, Matsumoto K and Nakamura T (1992) Lung may have an endocrine function producing hepatocyte growth factor in response to injury of distal organs. *Biochem Biophys Res Commun* **182**: 802–809

Yoshinaga Y, Fujita S, Gotoh M, Nakamura T, Kikuchi M and Hirohashi S (1992) Human lung cancer cell-line producing hepatocyte growth factor/scatter factor. *Jpn J Cancer Res* **83**: 1257–1261

Zarnegar R, Muga S, Rahija R and Michalopoulous GM (1990) Tissue distribution of hepatopoietin-A: a heparin-binding polypeptide growth factor for hepatocytes. *Proc Natl Acad Sci USA* **87**: 1252–1256

© Cancer Research Campaign 1998